# Management of retinopathy of prematurity (ROP) in a Polish cohort of infants

**DOI:** 10.1038/s41598-021-83985-5

**Published:** 2021-02-25

**Authors:** Anna Chmielarz-Czarnocińska, Marta Pawlak, Dawid Szpecht, Aneta Choręziak, Marta Szymankiewicz-Bręborowicz, Anna Gotz-Więckowska

**Affiliations:** 1 Department of Ophthalmology, Poznan University of Medical Sciences, Poznan, Poland; 2 Department of Neonatology, Poznan University of Medical Sciences, Poznan, Poland

**Keywords:** Paediatric research, Neonatology, Preterm birth, Retinopathy of prematurity, Epidemiology

## Abstract

Retinopathy of prematurity (ROP) is potentially blinding, but screening and timely treatment can stop its progression. The data on treatment outcomes of ROP from Central and Eastern Europe are scarce. Therefore, we aimed to analyze the latest results of ROP management in Poznan medical center to update the data from this world region. In the years 2016–2019, 178 patients (350 eyes) received treatment for ROP (6.1% of the screened population). The mean gestational age was 26 weeks (range 22–31 weeks), the mean birth weight was 868 g (range 410–1890 g). The most frequent ROP stage at treatment was zone II, stage 3 + (34.9%). As the first line of treatment, 115 infants (226 eyes, 64.6%) underwent laser photocoagulation (LP); 61 infants (120 eyes, 34.3%) received intravitreal ranibizumab injections (IVR); and 2 infants (4 eyes, 0.6%) were treated simultaneously with LP and IVR. One hundred twenty-six eyes (36%) of 63 patients required retreatment: 20.4% treated with LP and 66.7% treated with IVR. Retinal detachment occurred in 14 eyes (4%). The incidence of ROP, ROP requiring treatment, and reoccurrence rates are higher in the Polish population than in Western Europe and the USA. The identified treatment patterns find increasing use of anti-VEGF agents.

## Introduction

The research on retinopathy of prematurity (ROP) since its first description by Terry in 1942^1^ has uncovered much of the disease’s pathophysiology. However, its treatment remains a challenge, especially in extremely premature (EP; defined by the WHO as gestational age [GA] < 28 weeks and birth weight [BW] < 1000 g) or even smaller infants with GA < 25 weeks and BW < 750 g, who tend to develop its most aggressive forms. In Poland, approximately 400,000 children are born every year, of which 7% are born preterm, 0.3% with extremely low BW below 1000 g, and GA below 28 weeks^2^.

Interventions for severe ROP requiring treatment in high- and middle-income countries such as Poland include laser photocoagulation (LP) of the peripheral avascular retina, anti-vascular endothelial growth factor (anti-VEGF) injections and rarely used cryotherapy, and vitrectomies or scleral buckling for retinal detachment in the most advanced stages of the disease. Treatment prior to vitreoretinal traction is usually successful, but once a detachment occurs, the visual results are often quite poor^3^. Therefore, appropriate screening and timely, accurate treatment are crucial to stop the disease’s progression and prevent visual function deficits in ROP patients.

The data on treatment outcomes of ROP from Central and Eastern Europe are scarce, and no central registry for ROP patients exists in Poland. Therefore, the purpose of this study was to analyze the latest results of ROP treatment in Poznan medical center and to evaluate treatment paradigms in order to update the information on the outcomes of ROP management in a Central and Eastern European population.

## Patients and methods

This is a retrospective study of the results of ROP screening and treatment at a single academic center—the Gynecology and Obstetrics Hospital of Poznań University of Medical Sciences—from 1 January 2016 to 31 December 2019. The data of all patients screened and treated were abstracted from patients’ medical records and analyzed.

The Gynecology and Obstetrics Hospital of Poznan Medical University in Poland is a tertiary reference hospital, the largest obstetrics hospital in Poland, with approximately 7000 births a year. In its neonatal intensive care unit (NICU), all infants from two large regions of Poland (Greater Poland and Lubusz region) are treated for advanced ROP (approximately 12% of all children born in Poland are born in these two regions per year—48,245 out of a total of 388,178 births in the year 2018)^2^.

The study documents the outcomes of ROP management in two groups of patients treated in the NICU. The first group consisted of the patients born and treated in the Gynecology and Obstetrics Hospital of Poznań University of Medical Sciences (inborn). The second group comprised the patients from other medical centers who were screened for ROP by ophthalmologists in different hospitals but were transferred to the Poznan medical center to receive treatment due to severe ROP (outborn). These patients were born in two large regions of Poland (Greater Poland and Lubusz regions).

The screening examination followed the guidelines of the Polish Society of Ophthalmology for ROP screening (≤ 33 weeks of GA, ≤ 1800 g of BW, or if they were determined to be at high risk by a neonatologist)^4^. Patients were examined with binocular indirect ophthalmoscopy by a team of three trained pediatric ophthalmologists specializing in ROP. The screening was performed under topical anesthesia with proxymetacaine after pupil dilation with tropicamide 1% and phenylephrine 2.5% drops applied three times. The initial fundus examination was performed four weeks postnatally, with further follow-up every 7–10 days during the stay in the hospital or every 1–3 weeks depending on the advancement of the changes after the discharge of the patient from the hospital (until full vascularization of the retina, stabilization of the changes, or the necessity for treatment). Findings were classified according to the Revised International Classification of Retinopathy of Prematurity (ICROP)^5^.

The treatment criteria were based on the Early Treatment for Retinopathy of Prematurity (ETROP) guidelines^6^. Some patients were included past acute-phase treatment criteria as defined by ETROP on the decision of the examining ophthalmologist.

Treatment was provided within 72 h of detection of severe ROP. The initial treatment was either LP of the avascular retina or intravitreal anti-VEGF injection of ranibizumab (IVR) determined by the treating ophthalmologist depending on the severity of the disease. Anti-VEGF injections were preferred in the patients with zone I ROP with plus disease, in zone I ROP3 without plus disease, and in aggressive posterior retinopathy of prematurity (APROP).

Peripheral retinal ablations were carried out with a diode laser of a wavelength of 810 nm with confluent burns (Iris Medical OcuLight SL). Anti-VEGF injections were performed using a standard protocol with a dosage of 0.25 mg/0.025 mL ranibizumab (half the adult dose). The off-label use (until 2019—on 4 September 2019, ranibizumab received approval for ROP treatment in the EU from the European Commission—European Medicines Agency) of ranibizumab and the potential risks were discussed with the guardians of the patients, and their written informed consent was obtained.

Both procedures were performed under general anesthesia. The patients were examined the day after the procedure and every 7–10 days until the total regression of ROP was ascertained, changes were stable, or retreatment was necessary. All treated infants had a follow-up with the treating team.

The study followed the tenets of the Declaration of Helsinki and was approved by the Bioethics Committee of the Medical University in Poznań (Resolution No. 132/18).

Statistical analysis was performed with Statistica version 10, 2011 (Stat Soft, Inc., Tulsa, Oklahoma, United States) with Kolmogorov–Smirnov and Kruskal–Wallis tests. A P value of less than 0.05 was considered to indicate statistical significance.

## Results

### Patient cohort

A total of 1772 patients born in the years 2016–2019 in the Gynecology and Obstetrics Hospital of Poznań University of Medical Sciences were eligible for ROP screening according to Polish screening criteria. One hundred seventy-eight patients (350 eyes) received treatment for ROP: 108 patients (212 eyes) born and treated in our medical center (inborn) and 70 patients (138 eyes) who received primary neonatal care at other hospitals (outborn).

ROP occurred in 1.6% (459/29,304) of all inborn patients and in 25.9% (459/1772) of patients who underwent ROP screening in our medical center during the study period. The incidence of ROP requiring treatment in inborn patients was 23.5% (108/459) of all infants diagnosed with ROP, 6.1% (108/1772) of all infants screened. The specific numbers for particular years and total numbers of patients and eyes are presented in Table 1.

**Table 1:** Data on the number of patients/eyes in particular years.

	Number of patients (eyes)				
	2016	2017	2018	2019	total
Born*	7824	7494	7052	6934	29,304
Screened*	464	453	412	443	1772
With ROP*	86	135	122	116	459
**With ROP requiring treatment**	41 (82)	42 (78)	45 (89)	50 (100)	178 (350)
Inborn patients	23 (46)	30 (56)	23 (46)	32 (64)	108 (212)
Outborn patients	18 (36)	12 (23)	22 (43)	18 (36)	70 (138)

*Data for inborn patients.

**Figure 1:**
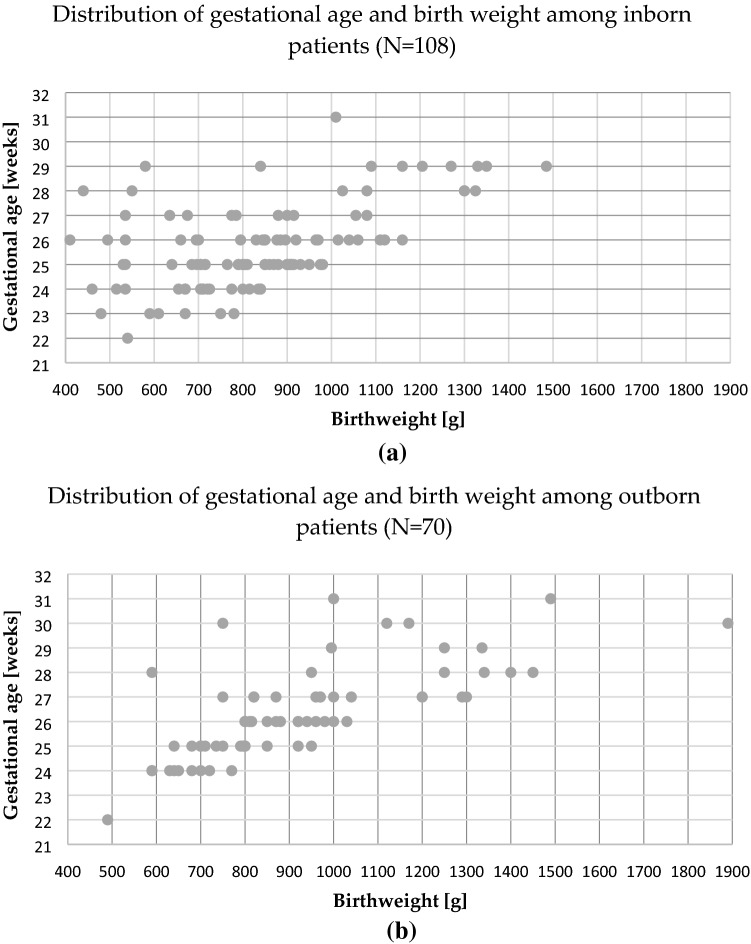
Distribution of gestational age and birth weight among: (**a**) inborn; (**b**) outborn patients requiring treatment, in the years 2016–2019.

For treated patients in the study period, the mean GA was 26 ± 2 weeks (SD), ranging from 22 to 31 weeks. Mean BW was 868 g ± 236, ranging from 410 to 1890 g. Distributions of GA and BW among children requiring treatment are shown in Fig. 1a for inborn patients and Fig. 1b for outborn infants. The mean BW of inborn patients was 836 g ± 216 and mean GA 26 weeks ± 1.7, and the mean BW of outborn infants was 918 g ± 257 and mean GA 26 weeks ± 1.9.

There was a statistically significant difference between inborn and outborn neonates in GA (mean GA 25.7 weeks vs 26.3 weeks; p = 0.018); however, there was no significant difference in BW between the groups. The statistical analysis of particular variables in particular years showed statistically significant differences in BW (mean BW 919 g vs 757 g; p = 0.099) and GA (mean 26.4 weeks vs 25.2 weeks; p = 0.014) in the group of inborn patients between years 2017 and 2019 and in BW in the group of treated patients in general (mean BW 927 g vs 801 g; p = 0.025) between the years 2017 and 2019. There were no statistical differences in BW and GA of outborn patients over the years.

### ROP characteristics at the time of treatment decision

The stage, zone, and presence or absence of plus disease frequencies of 212 eyes of 108 inborn patients are listed in Table 2.

**Table 2:** ROP advancement at the time of treatment decision in inborn patients, in the years 2016–2019.

Zone	Stage	Plus disease	Eyes (n = 212)	%
APROP			22	10.4
I	1	+	8	3.8
	2	−	2	0.9*
		+	4	1.9
	3	−	2	0.9
		+	8	3.8
II	1	−	2	0.9*
		+	9	4.2*
	2	−	13	6.1*
		+	43	20.3
	3	−	25	11.8*
		+	74	34.9

* treatment applied outside ETROP criteria, on the decision of ophthalmologist.

The most prevalent treatment indication was ROP 3 + in zone II, which was found in 34.9% of the eyes. During this study, clinicians treated 24% of eyes that did not meet ETROP treatment criteria, 31.4% in the years 2016 and 2017 and 17.3% in the years 2018 and 2019.

APROP occurred in 12 patients (22 eyes) born only at or before GA of 27 weeks (mean 25 ± 1; range 23–27) with BW of or lower than 1080 g (mean 778 ± 184; range 410–1080). There was a statistically significant difference in GA (24.7 weeks vs 25.9 weeks; p = 0.018) but not in BW between infants with ROP in zone I and zone II.

### ROP treatment

**Figure 2:**
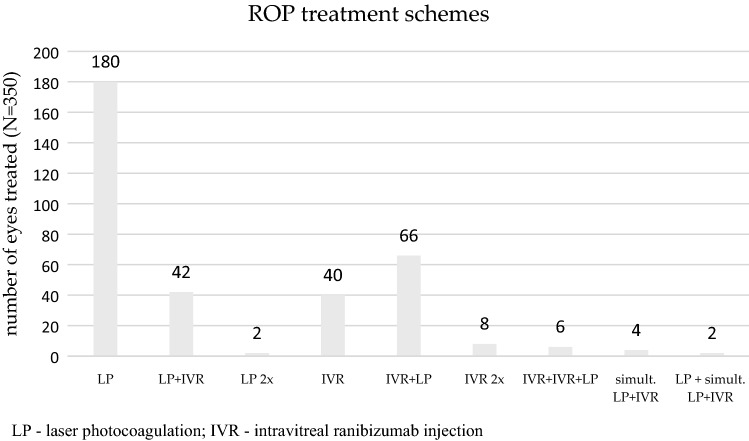
Distribution of treatment modalities for ROP patients in the years 2016–2019.

Treatment for ROP was administered bilaterally in 172 and unilaterally in 6 patients. Figure 2 shows the treatment modalities for all 178 patients (both inborn and outborn).

Three ROP specialists performed altogether 247 treatment sessions in 178 infants. As the first line of treatment, 115 infants (226 eyes, 64.6%) underwent photocoagulation of the retina with a diode laser in one or both eyes, 61 infants (120 eyes, 34.3%) were given intravitreal injections of ranibizumab, and 2 infants (4 eyes, 0.6%) were treated simultaneously with LP and IVR.

In zone I, the first line of treatment for ROP in inborn patients was IVR in 43 out of 46 interventions, while for the eyes with zone II disease, LP was performed in 122 out of 166 interventions. All inborn patients with APROP were treated initially with IVR.

There was no statistically significant difference between inborn and outborn neonates in postnatal age—PNA (mean 10 weeks vs 9 weeks) and postmenstrual age—PMA (mean 35 vs 36 weeks) at treatment.

**Figure 3:**
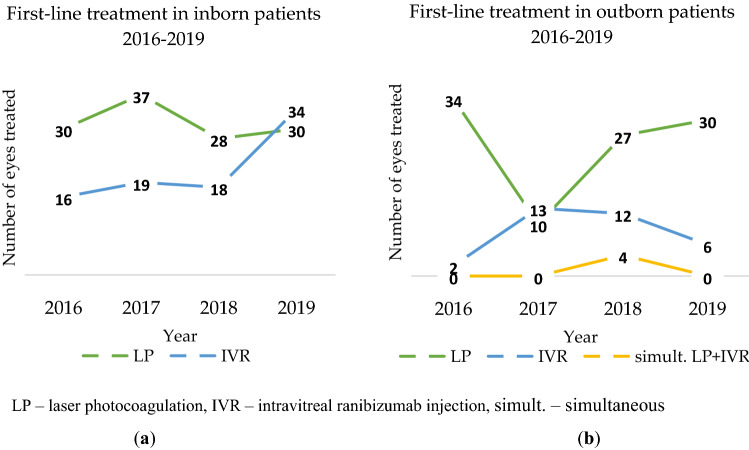
First-line treatment: (**a**) in inborn patients in the years 2016–2019; (**b**) in outborn patients in the years 2016–2019.

In inborn patients, laser ablation was the most frequently chosen first-line treatment method until the year 2019, when ranibizumab injections prevailed (see Fig. 3a). In outborn patients after 2017 when IVR was more frequently used, LP was a dominant first-line treatment method in the years 2018 and 2019 (see Fig. 3b).

### Retreatment

Due to the recurrence or persistence of active ROP, 126 eyes (36%) of 63 patients required retreatment: 46 out of 226 eyes (20.4%) treated in the first stage with laser therapy and 80 out of 120 eyes (66.7%) treated with ranibizumab injections. Four eyes treated simultaneously with LP and IVR and did not require retreatment. The number of retreatments needed after initial LP dropped to 13.3%, but increased after initial IVR to 70% in the year 2019. Retreatment modalities are shown in Fig. 2. The median interval between baseline treatment and first retreatment was 104 days (range 56–178) in the inborn patients’ group and 86 days (range 51–153) in the outborn patients’ group.

### Retinal detachment

Progression to retinal detachment was observed in 14 eyes (4% of eyes treated) of 7 patients (3 inborn and 4 outborn). Eleven eyes of 7 patients were qualified for vitrectomy as a salvage therapy because the disease progressed to the stages with partial retinal detachment (stages 4A and 4B). Vitrectomies were performed by a single vitreoretinal surgeon. After the surgeries, 8 eyes (72.7%) attained full or partial retinal reattachment. Three eyes were not qualified for vitrectomy due to advancement of the changes (stage 5). In the years 2018 and 2019, none of the inborn patients progressed to stage 4 or 5 of ROP.

## Discussion

This study provides an overview of ROP incidence and treatment in one of the biggest Polish ROP treatment centers. It analyses very recent data of the infants from two large regions of Poland where around 12% of Polish children are born. All who required treatment due to ROP were transferred to the Gynecology and Obstetrics Hospital of Poznań University of Medical Sciences.

The numbers of screened infants, patients with ROP, and patients with ROP requiring treatment were stable over the study period, despite the statistically significant difference in BW and GA in patients born in the years 2017 and 2019. This might result of still improving obstetrical and neonatal care in Poland, especially with better management and monitoring of supplemental oxygen use. PNA and PMA at treatment showed no significant differences during the study period, which, as stated in the analysis of the Swedish national quality registry (SWEDROP), probably points to a stable natural course of ROP^7^, or the period of the study was too short to note changes.

Our study group consisted of two populations: inborn patients who underwent ROP screening and treatment by the same team from the first screening examination with a continuous follow-up until the necessity of the treatment or the final screening examination, and an outborn cohort born outside our medical center who were screened in their place of birth and were only transferred for treatment by our ROP team after the examination. A significant difference in GA between inborn and outborn patients likely indicates slightly worse care provided outside the tertiary reference unit. However, no significant differences in PNA and PMA at treatment between inborn and outborn patients suggest a good quality of screening and timely decision on the transfer to our ROP treatment center.

ROP was found in 25.9% of screened infants, of which 6.1% required treatment, 4% of treated eyes developed an end-stage ROP (stage 4 or 5). Our results of the management and treatment of ROP are similar in some aspects but still differ in others from the results of more developed Western countries. For comparison, see Table 3.

**Table 3:** The incidence of ROP, screening criteria, gestational age, and birth weight of infants in the present study and the data from Western European countries and the USA.

Site of study	Study period	ROP diagnosed (%)	Treatment requiring ROP (%)	General screening criteria (week; g)	Mean GA (max)	Mean BW (max)
Poland (this study)	2016–2019	25.9	6.1	≤ 33; ≤ 1800	26 (31)	868 (1890/1490)
Germany^8,9^	2011–2015		3.0	< 32; < 1500	25	690
Netherlands^10,11^	2010–2016	19.2	5.2	< 31	25.2/26.2	715/730
Sweden^7^	2008–2012	30.3	5.2	< 31	24.5 (29)	702 (1230)
UK^12^	2013–2014		4.0	< 31; < 1251	25	706
USA^13^	2006–2011	43.1	6.9	≤ 30; ≤ 1500	28 (35)	1100 (3000)

A relatively low percentage of both infants with ROP and infants treated in our study probably results from a greater number of infants screened in Poland, as Polish screening criteria include infants born ≤ 33 weeks GA. Mean GA at birth and BW of the treated infants in our study were higher than in more developed European countries but lower than in the USA. However, in the study by Quinn et al., only 0.75% of treated American infants with BW of 1501 g or more and GA of older than 30 weeks developed severe ROP^13^. It shows that neonatal care in Poland, although improving, is still less advanced than in Western European countries and the USA.

In our study, no infant born after 31 weeks of GA (inborn or outborn) required treatment for ROP. A more conclusive national or at least multi-center analysis would be necessary to answer the question if Polish screening guidelines (which include infants ≤ 33 weeks of GA) do not cover too many children.

Patients with type 1 ROP accounted for 76% of the cases in our study, meaning that 24% fell outside criteria for treatment listed by the ETROP. Treating milder forms of ROP, however, is also common in other countries. Type 1 ROP constituted 83.2%^7^, 62.39%^12^, and 89.3%^13^ of all treated infants in Sweden, the UK, and the USA, respectively. As suggested by Slidsborg et al.^14^ in a Danish study, various organizational reasons, such as long-distance transport, may contribute to an occasional lack of adherence to treatment criteria. Additionally, plus disease, which is a major criterion for type 1 ROP, is a very subjective and selective diagnosis^7,15^. Therefore, sometimes children classified with “pre-plus” disease rather than “plus disease” are treated as well. Walz et al.^9^ point to the fact that in bilateral cases where only one eye requires immediate treatment and the other has an active disease of slightly less severity, it may be sensible to treat both eyes in one setting to save the infant two procedures under general anesthesia only a few days apart. We noticed, however, a strong trend towards better adherence to the international standards over the study period: in the years 2016 and 2017, 68.6% compared to 82.7% of children in the years 2018 and 2019 were treated according to ETROP criteria.

The most frequent treatment indication in our study, similarly to other countries, was ROP 3 + in zone II. In our cohort, this stage of advancement was present in 74 eyes (34.9%), in Germany in 78.8%^9^, and in the USA in 68.4%^13^. APROP, the most sight-threatening indication for treatment, was more common in our cohort than in other countries. It was found in 22/212 eyes (in 10.4% of the eyes of inborn patients), in Germany in 6.2%, in Sweden in 8.3%, and in the USA in 3.3%.

The gold standard for ROP treatment worldwide is still LP of the avascular retina^3,16^. However, since 2011, the publication year of the BEAT-ROP study^17^, an increasing trend of using anti-VEGF agents as first-line monotherapy could be noticed. Their advantages include easier administration than laser, fast regression of neovascularization and plus disease, perhaps the preservation of the visual field^17^, and less myopia^18^ in long-term observation. A much-debated question is whether and to what extent anti-VEGF agents applied locally may affect the overall development of children^19–23^. Intravitreal injections also require longer and more frequent follow-up^24^ and introduce a risk of retinal abnormalities in the future^25,26^. Yet, promising results of this treatment and lack of solid proofs of its adverse systemic and ophthalmological side effects make it a possible supplementary as well as first-line treatment option, at least in some clinical cases. A broad, multi-center RAINBOW study’s conclusion was that ranibizumab at 0.2 mg might be superior to laser therapy^27^.

The unsatisfactory results of ROP treatment in our medical centre compared to other centers in more developed countries^28^ prompted us to change the treatment regimen of ROP in the year 2015 from diode LP of the retina in all patients with ROP requiring treatment followed by a second course of laser or an anti-VEGF injection treated as salvage therapy in the most challenging cases. From November 2015, a more individualized approach was issued, and anti-VEGF injections began to be a first-line treatment in chosen patients. The first vitrectomy in a preterm neonate financed by the Polish National Health Fund took place in Poznan medical centre on 29 April 2016. Since then, our medical centre has been performing vitrectomies as a reference centre for the whole of Poland. Previously, this procedure for Polish preterm infants was only available commercially in a single private health care centre in Poland or abroad.

We concluded that the most common reason for the failure of our treatment before the year 2016 was insufficient laser therapy in APROP and in the zone I ROP; therefore, we changed the treatment strategy for anti-VEGF injection as a first-line treatment followed, in chosen cases, by supplementary laser therapy. In our opinion, this scheme is a better solution than laser therapy in APROP, which usually requires dividing the procedure into at least two stages (difficult photocoagulation of the central retina), which is associated with general anaesthesia and dangerous photocoagulation close to the macula. In our study, there were no increased short-term complications reported with the use of anti-VEGF. However, an open problem remains the follow-up period in children after injections without laser ablation. As concluded in the American Academy of Ophthalmology report on primary treatment with anti-VEGF in type 1 ROP, most zone I eyes treated with anti-VEGF may never completely vascularize and may still need retreatment after 55 weeks’ PMA^24^.

In the present study, 34.3% of 178 patients had intravitreal injections of ranibizumab as a first-line treatment. It is a relatively high percentage and one comparable with German anti-VEGF monotherapy in 32% of patients in 2014^8^. In other studies, the vast majority of patients had laser therapy only—90% in the SWEDROP study^7^ and 90.5% in the UK^12^—emphasizing the good effect of this treatment. However, the studies analyze the treatment of ROP in the years 2008–2012 and 2013–2014, respectively, when anti-VEGF was not so widely used. In experts’ opinion, an adverse outcome in the first few months after laser treatment is due to inadequate treatment or smoldering disease activity, but not to disease reactivation^29^. In our study, the necessity of retreatment after the laser was quite high (20.4%), but the retreatment rate dropped over the years, which most likely indicates a better quality of treatment with laser provided by our treating team. The number of retreatments after anti-VEGF therapy in our study was high (66.7%). It likely results from more advanced changes in infants with APROP and ROP in zone I, but also confirms the need for careful and long-term follow-up of patients treated with intravitreal injections.

Of the treated eyes, 4% progressed into ROP stages 4 and 5, but satisfactory anatomical results were achieved in 72.7% of eyes treated. Since follow-up information is lacking for the time being, we are unable to report on the visual outcomes. In the Swedish study, 6% of infants had a retinal detachment in at least one eye.

This study, the largest analysis of a Polish ROP cohort, reveals possible areas of improvement in the management of ROP, including better cooperation with lower reference centers, enabling therapy to be performed at the optimal time. At present, ROP incidence, severity, and reoccurrence rates in Poland are higher than in more developed countries of Western Europe and the USA. Revisiting Polish screening criteria could be considered, but only after a national or a multi-centre analysis of ROP prevalence.

## Data Availability

The datasets generated during and/or analyzed during the current study are available from the corresponding author on reasonable request.
